# Comparative profiling of *agr* locus, virulence, and biofilm-production genes of human and ovine non-*aureus* staphylococci

**DOI:** 10.1186/s12917-022-03257-w

**Published:** 2022-06-02

**Authors:** Elisa Azara, Carla Maria Longheu, Sonia Attene, Silvana Sanna, Marco Sale, Maria Filippa Addis, Sebastiana Tola

**Affiliations:** 1 Istituto Zooprofilattico Sperimentale della Sardegna “G. Pegreffi”, Via Vienna 2, 07100 Sassari, Italy; 2 Ospedale “San Francesco”, 08100 Nuoro, Italy; 3 Azienda Ospedaliera Universitaria, 07100 Sassari, Italy; 4 Ospedale “A. Segni”, 07014 Ozieri, Italy; 5 Dipartimento di Medicina Veterinaria, Università degli Studi di Milano, 26900 Lodi, Italy

**Keywords:** Non-*aureus* staphylococci, Human, Ovine, Biofilm, Adhesins, Toxins, *Quorum sensing*

## Abstract

**Background:**

In a collaboration between animal and human health care professionals, we assessed the genetic characteristics shared by non-*aureus* staphylococci (NAS) infecting humans and dairy ewes to investigate their relatedness in a region concentrating half of the total National sheep stock. We examined by PCR 125 ovine and 70 human NAS for biofilm production, pyrogenic toxins, adhesins, autolysins genes, and accessory gene regulator (*agr*) locus. The microtiter plate assay (MPA) was used for the phenotypic screening of biofilm production. Ovine NAS included *S. epidermidis*, *S. chromogenes*, *S. haemolyticus*, *S. simulans*, *S. caprae*, *S. warneri*, *S. saprophyticus*, *S. intermedius*, and *S. muscae*. Human NAS included *S. haemolyticus*, *S. epidermidis*, *S. hominis*, *S. lugdunensis*, *S. capitis*, *S. warneri*, *S. xylosus*, *S. pasteuri*, and *S. saprophyticus* subsp. *bovis*.

**Results:**

Phenotypically, 41 (32.8%) ovine and 24 (34.3%) human isolates were characterized as biofilm producers. Of the ovine isolates, 12 were classified as biofilm-producing while the remaining 29 as weak biofilm-producing. All 24 human isolates were considered weak biofilm-producing. Few *S. epidermidis* isolates harbored the *icaA/D* genes coding for the polysaccharide intercellular adhesin (PIA), while the *bhp*, *aap,* and *embp* genes coding biofilm accumulation proteins were present in both non-producing and biofilm-producing isolates. Fifty-nine sheep NAS (all *S. epidermidis*, 1 *S. chromogenes,* and 1 *S. haemolyticus*) and 27 human NAS (all *S. epidermidis* and 1 *S. warneri*) were positive for the *agr* locus: agr-3se (57.8%) followed by agr-1se (36.8%) predominated in sheep, while agr-1se (65.4%), followed by agr-2se (34.6%) predominated in humans.

Concerning virulence genes, 40, 39.2, 47.2%, 52.8, 80 and 43.2% of the sheep isolates carried *atlE*, *aae*, *sdrF*, *sdrG*, *eno* and *epb*S respectively, against 37.1, 42.8, 32.8, 60, 100 and 100% of human isolates. Enterotoxins and *tsst* were not detected.

**Conclusions:**

Considerable variation in biofilm formation ability was observed among NAS isolates from ovine and human samples. *S. epidermidis* was the best biofilm producer with the highest prevalence of adhesin-encoding genes.

**Supplementary Information:**

The online version contains supplementary material available at 10.1186/s12917-022-03257-w.

## Background

Half of the total Italian dairy sheep stock is farmed in Sardinia, an island located in the Mediterranean Sea. Sardinia has approximately 3.5 million dairy sheep, with a human population of around 1.6 million inhabitants. Accordingly, a relevant part of the regional economy relies on dairy sheep farming, and controlling intra-mammary infections (IMI) is crucial.

Sheep mastitis prevalence is estimated to range from 5 to 30%, and several reports indicate that non-*aureus* staphylococci (NAS) are the most prevalent microrganisms causing subclinical disease in small ruminants [1–4]. Therefore, the exchange of colonizing and pathogenic microorganisms, with their antimicrobial-resistance and pathogenicity gene pools, can occur among sheep and farmers. NAS have recently gained attention as nosocomial agents causing frequent infections in debilitated or compromised patients, mainly associated with catheters and other indwelling medical devices [5]. NAS, and in particular *S. epidermidis*, can produce a multicellular biofilm that decreases the antibiotic concentration within the colony, promotes multiplication, and enhances the survival of invading bacteria [6]. Biofilm formation can be best assessed by the microtiter plate assay (MPA), as it produces a quantitative result by measuring the optical density of the stained biofilm [7, 8]. The main constituent of the NAS biofilm matrix is a linear 1,6-linked glycosaminoglycan, also known as polysaccharide intercellular adhesin (PIA), synthesized by proteins encoded by the intercellular adhesion (*ica*) operon. Among the *ica* genes, *ica*A and *ica*D have an essential role in biofilm production [9]. The coexistence of both *ica*A/D genes leads to the full phenotypic expression of the capsular polysaccharide [9]. However, PIA-independent biofilms involving accumulation-associated protein (Aap), biofilm homologue protein (Bhp) and extracellular matrix-binding protein (Embp) have also been reported [10, 11].

Generally, NAS can produce several virulence factors that contribute collectively to colonization and invasion of host cells and tissues, as well as evasion of immune responses [12]. Virulence factors include the autolysins AtlE and Aae [13, 14], and microbial surface components recognizing adhesive matrix molecules (MSCRAMMs) that mediate initial adhesion to different surfaces and promote colonization and serum protein binding [15]. The best known *S. epidermidis* MSCRAMMs are the fibrinogen-binding protein SdrG [16], and the collagen/keratin-binding protein SdrF [17, 18].

Furthermore, the production of various toxins can also contribute to NAS virulence [19], including staphylococcal enterotoxins (SEs) and toxic shock syndrome toxin 1 (TSST-1) [20]. Five serological types of SEs are typically known (SEA to SEE), but new types of SEs (SEG to SE1V) have also been identified and characterized [21, 22]. The *quorum-sensing* system (QS) *agr*, i.e. accessory gene regulator [23, 24] regulates biofilm formation, intercellular communication, and numerous virulence factors including toxins and autolysins. Three distinct genetic groups (types 1, 2, and 3) based on the *agr* locus polymorphism have been described in *S. epidermidis* [25], but data on the genetic polymorphisms of the *agr* locus in different species of NAS were not available in the scientific literature at the beginning of this investigation.

In this study, we compared the molecular characteristics of NAS isolated from the milk of sheep with mastitis and human clinical specimens with the following aims: 1) assess the biofilm production characteristics by phenotypic and genotypic methods, 2) carry out genotypic screening for a set of MSCRAMMs, autolysins, enterotoxins and *tsst*-1 genes and 3) investigate the *agr* locus and its genetic polymorphism.

## Results

### Ovine NAS

We analyzed a total of 125 isolates, including *S. epidermidis* (*n* = 57), *S. chromogenes* (*n* = 29), *S. haemolyticus* (*n* = 17), *S. simulans* (*n* = 8), *S. caprae* (*n* = 6), *S. warneri* (*n* = 5), *S. saprophyticus* (*n* = 1), *S. intermedius* (*n* = 1) and *S. muscae* (n = 1). Table 1 reports the isolates included in the study, while Supplementary Table S1 reports the primers used for PCR amplifications.

**Table 1:** Distribution of ovine and human NAS isolates according to the specimen origin

**Ovine NAS**^a^									
Source	*S. epi*	*S. chr*	*S. hae*	*S. sim*	*S. cpr*	*S. war*	*S. sap*	*S. int*	*S. mus*
Milk	57	29	17	8	6	5	1	1	1
**Human NAS**^a^									
Source	*S. hae*	*S. epi*	*S. lug*	*S. hom*	*S. cap*	*S. war*	*S. xyl*	*S. pas*	*S. sap*
Nasal swab	6	5	–	–	–	–	–	–	–
Blood	4	6	–	1	–	–	–	–	–
Skin swab	2	3	–	–	2	–	1	–	–
Pus	1	3	–	–	–	–	–	1	–
Peritoneal fluid	2	–	2	–	–	–	–	–	–
Seminal fluid	4	–	–	–	–	–	–	–	–
Injury	–	3	1	–	–	–	–	–	–
Ear swab	2	–	–	–	–	1	–	–	–
Oral swab	1	1	–	–	–	–	–	–	1
Urine	2	–	–	1	–	–	–	–	–
C.V.C^b^	–	2	–	–	–	–	–	–	–
Ulcer swab	–	1	–	–	–	–	–	–	–
F.V.C.^b^	–	1	–	–	–	–	–	–	–
Peritoneal swab	1	–	–	–	–	–	–	–	–
Vaginal swab	–	–	1	–	–	–	–	–	–
Glans swab	1	–	–	–	–	–	–	–	–
Pleural fluid	–	–	–	1	–	–	–	–	–
Fluid drainage	–	–	–	–	–	1	–	–	–
B.L. fluid^b^	1	–	–	–	–	–	–	–	–
N.P. aspirate^b^	–	–	–	–	1	–	–	–	–
Bile	1	–	–	–	–	–	–	–	–
Biopsy	–	1	–	–	–	–	–	–	–
Prosthesis	–	–	–	1	–	–	–	–	–
Total	28	26	4	4	3	2	1	1	1

^a^*Isolate abbreviations*: *S. hae*, *S. haemolyticus; S. epi, S. epidermidis; S. chr, S. chromogenes; S. sim, S. simulans; S. lug, S. lugdunensis; S. hom, S. hominis; S. cpr, S. caprae; S. S. cap, S. capitis; S. war, S. warneri; S. xyl, S. xylosus; S. pas, S. pasteuri; S.int, S. intermedius; S. sap, S. saprophyticus subsp. Bovis; S. mus, S. muscae*

^b^*Specimen abbreviations*: *C.V.C* central venous catheter, *F.V.C* femoral venous catheter, *B.L.fluid* bronchoalveolar lavage fluid, *N.P* aspirate, nasopharyngeal aspirate

Table 2 summarizes the biofilm formation results. Out of 125 isolates examined, 41 (32.8%) were classified as biofilm producers; of these 29 (23.2%) were classified as weak biofilm-producers (WBP) while 12 (9.6%) as biofilm-producers (BP). Only one isolate harbored both *ica*A and *ica*D genes while two had only the *ica*A gene. On the other hand, 37 (29.6%), 22 (17.6%) and 63 (50.4%) isolates harbored the *bhp*, *aap* and *embp* genes, respectively (Table 2). For autolysin genes, 50 (40%) isolates were PCR positive for *alt*E while 49 (39.2%) were positive for *aae*. Concerning adhesion factors (MSCRAMMs), 59 (47.2%), 66 (52.8%), 100 (80%) and 54 (43.2%) isolates harbored *sdr*F, *sdr*G, *eno* and *epb*S, respectively. All isolates were negative for *clf*A (Table 3). Regarding the *agr* type, 21 (16.8%) isolates belonged to *agr*-1 while 33 (26.4%) to *agr*-3_._ None of the isolates belonged to type 2 (Table 4). No amplification was obtained for the toxin genes analyzed.

**Table 2:** Phenotypic characterisation of biofilm production by MPA and genotypic detection by PCR of *ica*, *bhp*, *aap* and *embp* genes from 125 ovine and 70 human NAS isolates

**OVINE** Isolates (n)	Biofilm production (MPA)				Biofilm genes											
					PIA						Proteinaceous factors					
	*WBP*^a^		*BP*^b^		*ica*A		*ica*D		*ica*A/D		*bhp*		*aap*		*embp*	
	*n*	*%*	*n*	*%*	*n*	*%*	*n*	*%*	*n*	*%*	*n*	*%*	*n*	*%*	*n*	*%*
*S. epi* (57)	17	30	11	19	1	1.7	1	1.7	1	1.7	30	52.6	20	16	54	94.7
*S. chr* (29)	0	0	1	3.4	0	0	0	0	0	0	4	13.8	0	0	8	27.5
*S. hae* (17)	0	0	0	0	0	0	0	0	0	0	0	0	2	11.7	1	5.8
*S. sim* (8)	7	87.5	0	0	0	0	0	0	0	0	0	0	0	0	0	0
*S. cpr* (6)	3	50	0	0	1	16.6	0	0	0	0	1	16.6	0	0	0	0
*S. war* (5)	0	0	0	0	0	0	0	0	0	0	2	40	0	0	0	0
*S. sap* (1)	1	100	0	0	1	100	0	0	0	0	0	0	0	0	0	0
*S. int* (1)	1	100	0	0	0	0	0	0	0	0	0	0	0	0	0	0
*S. mus* (1)	0	0	0	0	0	0	0	0	0	0	0	0	0	0	0	0
**Total** (125)	**29**	23.2	**12**	9.6	**3**	2.4	**1**	0.8	**1**	0.8	**37**	29.6	**22**	17.6	**63**	50.4
**HUMAN** Isolates (n)	Biofilm production (MPA)				Biofilm genes											
					PIA						Proteinaceous factors					
	*WBP*^a^		*BP*^b^		*ica*A		*ica*D		*ica*A/D		*bhp*		*aap*		*embp*	
	*n*	*%*	*n*	*%*	*n*	*%*	*n*	*%*	*n*	*%*	*n*	*%*	*n*	*%*	*n*	*%*
*S. hae* (28)	1	3.6	0	0	0	0	0	0	0	0	0	0	4	14.3	0	0
*S. epi* (26)	9	34.6	0	0	10	38.5	10	38.5	10	38.5	16	61.5	25	96.1	23	88.5
*S. lug* (4)	3	75	0	0	0	0	0	0	0	0	0	0	0	0	0	0
*S. hom* (4)	4	100	0	0	0	0	0	0	0	0	0	0	4	100	0	0
*S. cap* (3)	3	100	0	0	3	100	0	0	0	0	0	0	0	0	0	0
*S. war* (2)	2	100	0	0	0	0	0	0	0	0	0	0	1	50	1	50
*S. xyl* (1)	1	100	0	0	1	100	0	0	0	0	0	0	0	0	0	0
*S. pas* (1)	0	0	0	0	0	0	0	0	0	0	0	0	0	0	0	0
*S. sap* (1)	1	100	0	0	0	0	0	0	0	0	0	0	0	0	0	0
**Total** (70)	**24**	34.3	0	0	**14**	20	**10**	14.3	**10**	14.3	**16**	22.8	**34**	48.6	**24**	34.3

*Isolate abbreviations*: *S. hae, S. haemolyticus; S. epi, S. epidermidis; S. chr, S. chromogenes; S. sim, S. simulans; S. lug, S. lugdunensis; S. hom, S. hominis; S. cpr, S. caprae; S. S. cap, S. capitis; S. war, S. warneri; S. xyl, S. xylosus; S. pas, S. pasteuri; S.int, S. intermedius;S. sap, S. saprophyticus subsp. bovis; S. mus, S. muscae*

^a^*WBP weak biofilm-producing* isolate

^b^*BP biofilm-producing* isolate

**Table 3:** Results of testing 125 ovine and 70 human NAS isolates for autolysins and MSCRAMMS genes by PCR

**OVINE** Isolates (n)	Autolysins				MSCRAMMS																					
	*atlE*		*aae*		*sdr*F		*sdr*G		*clfA*		*clfB*		*fnbA*		*fnbB*		*bbp*		*cna*		*fib*		*eno*		*epbS*	
	*n*	*%*	*n*	*%*	*n*	*%*	*n*	*%*	*n*	*%*	*n*	*%*	*n*	*%*	*n*	*%*	*n*	*%*	*n*	*%*	*n*	*%*	*n*	*%*	*n*	*%*
*S. epi* (57)	48	84.2	46	80.7	45	78.9	50	87.7	0	0	0	0	0	0	0	0	0	0	0	0	0	0	56	98	52	91
*S. chr* (29)	1	3.4	0	0	9	31	13	22.8	0	0	2	7	2	7	1	3.4	0	0	2	7	2	7	16	55	2	7
*S. hae* (17)	1	5.8	1	5.8	1	5.8	1	5.8	0	0	0	0	0	0	0	0	1	6	0	0	0	0	15	88	0	0
*S. sim* (8)	0	0	1	12.5	0	0	0	0	0	0	0	0	0	0	0	0	0	0	0	0	0	0	0	0	0	0
*S. cpr* (6)	0	0	0	0	1	16.6	0	0	0	0	0	0	0	0	0	0	0	0	0	0	0	0	6	100	0	0
*S. war* (5)	0	0	1	20	3	60	2	40	0	0	0	0	0	0	0	0	0	0	0	0	0	0	4	80	0	0
*S. sap* (1)	0	0	0	0	0	0	0	0	0	0	0	0	0	0	0	0	0	0	0	0	0	0	1	100	0	0
*S. int* (1)	0	0	0	0	0	0	0	0	0	0	0	0	0	0	0	0	0	0	0	0	0	0	1	100	0	0
*S. mus* (1)	0	0	0	0	0	0	0	0	0	0	0	0	0	0	0	0	0	0	0	0	0	0	1	100	0	0
**Total** (125)	**50**	40	**49**	39.2	**59**	47.2	**66**	52.8	**0**	0	**2**	1.6	**2**	1.6	**1**	0.8	**1**	0.8	**2**	1.6	**2**	1.6	**100**	80	**54**	43.2
**HUMAN** Isolates (n)	Autolysins				MSCRAMMS																					
	*atlE*		*aae*		*sdr*F		*sdr*G		*clfA*		*clfB*		*fnbA*		*fnbB*		*bbp*		*cna*		*fib*		*eno*		*epbS*	
	*n*	*%*	*n*	*%*	*n*	*%*	*n*	*%*	*n*	*%*	*n*	*%*	*n*	*%*	*n*	*%*	*n*	*%*	*n*	*%*	*n*	*%*	*n*	*%*	*n*	*%*
*S. hae* (28)	0	0	0	0	0	0	0	0	0	0	0	0	0	0	0	0	0	0	0	0	0	0	28	100	0	0
*S. epi* (26)	25	96.1	26	100	21	80.8	26	100	0	0	0	0	0	0	0	0	0	0	0	0	0	0	26	100	26	100
*S. lug* (4)	0	0	0	0	0	0	4	100	0	0	0	0	0	0	0	0	0	0	0	0	0	0	4	100	0	0
*S. hom* (4)	0	0	0	0	0	0	4	100	0	0	0	0	0	0	0	0	0	0	0	0	0	0	4	100	0	0
*S. cap* (3)	0	0	0	0	0	0	3	100	0	0	0	0	0	0	0	0	0	0	0	0	0	0	3	100	0	0
*S. war* (2)	1	50	1	50	1	50	2	100	1	50	0	0	0	0	0	0	0	0	0	0	0	0	2	100	0	0
*S. xyl* (1)	0	0	1	100	1	100	1	100	0	0	0	0	0	0	0	0	0	0	0	0	0	0	1	100	0	0
*S. pas* (1)	0	0	1	100	0	0	1	100	0	0	0	0	0	0	0	0	0	0	0	0	0	0	1	100	0	0
*S. sap* (1)	0	0	1	100	0	0	1	100	0	0	0	0	0	0	0	0	0	0	0	0	0	0	1	100	0	0
**Total** (70)	**26**	37.1	**30**	42.8	**23**	32.8	**42**	60	**1**	50	**0**	0	**0**	0	**0**	0	**0**	0	**0**	0	**0**	0	**70**	100	**26**	100

*Isolate abbreviations*: *S. hae, S. haemolyticus; S. epi, S. epidermidis; S. chr, S. chromogenes; S. sim, S. simulans; S. lug, S. lugdunensis; S. hom, S. hominis; S. cpr, S. caprae; S. S. cap, S. capitis; S. war, S. warneri; S. xyl, S. xylosus; S. pas, S. pasteuri; S.int, S. intermedius;S. sap, S. saprophyticus subsp. bovis; S. mus, S. muscae*

**Table 4:** Results of testing 125 ovine and 70 human NAS isolates for *agr* locus by PCR

**OVINE** Isolates (n)	***agr *locus**							
	*agr* 200 bp		Type 1		Type 2		Type 3	
	*n*	*%*	*n*	*%*	*n*	*%*	*n*	*%*
*S. epi* (57)	57	100	21	36.8	0	0	33	57.8
*S. chr* (29)	1	3.4	0	0	0	0	0	0
*S. hae* (17)	1	5.8	0	0	0	0	0	0
*S. sim* (8)	0	0	0	0	0	0	0	0
*S. cpr* (6)	0	0	0	0	0	0	0	0
*S. war* (5)	0	0	0	0	0	0	0	0
*S. sap* (1)	0	0	0	0	0	0	0	0
*S. int* (1)	0	0	0	0	0	0	0	0
*S. mus* (1)	0	0	0	0	0	0	0	0
**Total** (125)	**59**	47.2	**21**	16.8	**0**	0	**33**	26.4
**HUMAN** Isolates (n)	***agr *locus**							
	*agr* 200 bp		Type 1		Type 2		Type 3	
	*n*	*%*	*n*	*%*	*n*	*%*	*n*	*%*
*S. hae* (28)	0	0	0	0	0	0	0	0
*S. epi* (26)	26	100	17	65.4	9	34.6	0	0
*S. lug* (4)	0	0	0	0	0	0	0	0
*S. hom* (4)	0	0	0	0	0	0	0	0
*S. cap* (3)	0	0	0	0	0	0	0	0
*S. war* (2)	1	50	0	0	0	0	0	0
*S. xyl* (1)	0	0	0	0	0	0	0	0
*S. pas* (1)	0	0	0	0	0	0	0	0
*S. sap* (1)	0	0	0	0	0	0	0	0
**Total** (70)	**27**	38.6	**17**	24.3	**9**	12.8	**0**	0

*Isolate abbreviations: S. hae, S. haemolyticus; S. epi, S. epidermidis; S. chr, S. chromogenes; S. sim, S. simulans; S. lug, S. lugdunensis; S. hom, S. hominis; S. cpr, S. caprae; S. S. cap, S. capitis; S. war, S. warneri; S. xyl, S. xylosus; S. pas, S. pasteuri; S.int, S. intermedius;S. sap, S. saprophyticus subsp. bovis; S. mus, S. muscae*

#### *S. epidermidis*

*S. epidermidis* was the most represented ovine NAS. Out of 57 isolates, 17 (30%) were classified as WBP and 11 (19%) as BP; only one non-BP harbored both *ica*A/D genes. On the other hand, 54 (94.7%), 30 (52.6%) and 20 (16%) isolates harbored *embp*, *bhp* and *aap*, respectively. Concerning autolysin genes, 48 (84.2%) were PCR-positive for *atl*E and 46 (80.7%) for *aae*. MSCRAMM genes were found in high percentages: 98% for *eno*, 91% for *epb*S, 87.7% for s*df*G and 78.9% for *sdr*F. No amplification was obtained for *clf*A/B, *fnb*A/B, *bbp*, *cna* and *fib* (Table 3). All *S. epidermidis* isolates were positive for the *agr* locus: 33 (57.8%) belonged to *agr*-3_se_ while 21 (36.8%) to *agr*-1_se_ (Table 4). Three isolates were non-typeable.

#### *S. chromogenes*, *S. haemolyticus*, and minor ovine NAS

*S. chromogenes* (*n* = 29) and *S. haemolyticus* (*n* = 17) were the most prevalent species in ovine milk samples after *S. epidermidis*. Table 2 shows that only 1 *S. chromogenes* was classified as BP while 7 (87.5%) *S. simulans* and 3 (50%) *S. caprae* were classified as WBP. The details are reported in Table 2.

### Human NAS

The distribution of biofilm, autolysins, and MSCRAMMs genes in the 70 human NAS, including *S. haemolyticus*, *S. epidermidis, S. lugdunensis*, *S. hominis*, *S. capitis*, *S. warneri*, *S. xylosus*, *S. pasteuri* and *S. saprophyticus subsp. bovis* is shown in Tables 2 and 3*.*

#### *S. haemolyticus*

Out of 28 *S. haemolyticus* isolates examined by MPA, only 1 (3.6%) was classified as WBP. None of the *S. haemolyticus* isolates harbored *ica*A/D, *bhp* and *embp*. On the contrary, the WBP (from blood) and other 3 non-BP (from nasal swab, blood, and glans swab) isolates possessed the *aap* gene. No amplification was observed for atlE, aae, sdrF/G, *clf*A/B, *fnb*A/B, *bbp*, *cna, fib*, *epb*S, *agr* locus (Table 3), and toxin genes. However, all isolates harbored the *eno* gene.

#### *S. epidermidis*

Among the 26 *S. epidermidis* isolates, 9 (34.6%) were classified as WBP; 4 of them (2 from catheter and 2 from blood) harbored both *ica*A and *ica*D whereas the remaining 5 isolates were *ica*A/D negative. The other 5 *ica*A/D positive isolates, classified as non-BP, were also positive for the other biofilm genes analyzed. Out of 17 non-BP and *ica*A/D negative isolates, 3 (2 from pus and 1 from oral swab) harbored *aap* and *embp* while 1 (from skin swab) harbored all *bhp*, *aap,* and *embp* genes. Overall, 16 (61.5%), 25 (96.1%) and 23 (88.5%) isolates carried the *bhp*, *aap* and *embp*, respectively (Table 2). Data on the prevalence of autolysins and MSCRAMM genes by PCR are shown in Table 3. Concerning autolysin genes, all isolates were PCR positive for *aae* and almost all (25/26 = 96.1%) were positive for *alt*E. Regarding adhesion factors, all *S. epidermidis* isolates were positive for *sdr*G, *eno,* and *epb*S, while 21/26 (80.8%) for *sdr*F. In four of them (2 from pus, 1 from biopsy and 1 from skin swab), a PCR product smaller than the expected size was observed. Sequence analysis of these amplicons showed the absence of an 84 bp fragment. In contrast, no amplification was observed for *clf*A/B, *fnb*A/B, *bbp*, *cna*, and *fib*. Determination of the *agr* type was performed in all *S. epidermidis* isolates: 17 (65.4%) belonged to *agr*-1_se_ whilst 9 (34.6%) to *agr*-2_se_ (Table 4). Among the 17 agr-1_se_ isolates, only 3 (1 from femoral venous catheter, 1 from nasal swab and 1 from pus) carried simultaneously *ica*A/D, *bhp*, *aap*, *embp*, *atl*E, *aae*, *sdr*F *sdr*G, *eno,* and *epb*S, associated with biofilm formation. Of these, 1 was WBP and two non-BP. Among the 9 agr-2_se_ isolates, only 1 (from blood) possessed these genes and it was a WBP isolate. Regarding the pyrogenic toxin genes, amplification was not observed in the *S. epidermidis* isolates or the remaining staphylococci.

### Minor human NAS

Out of the 4 *S. lugdunensis* isolates examined, 3 (2 from peritoneal fluid and 1 from vaginal swab) were considered as WBP. However, they harbored only *sdr*G and *eno*.

All 4 *S. hominis* isolates were phenotypically WBP but were PCR-positivity only for *aap, sdr*G, and *eno*. All 3 WBP *S. capitis* isolates were PCR-positive for *ica*A, *sdr*G, and *eno*. Tables 3 and 4 report the PCR results for *S. warneri*, *S. xylosus*, *S. pasteuri*, and *S. saprophyticus* subsp. *bovis*. Of note, one WPB *S. warneri* isolate (from fluid drainage) was negative for *ica*A/D and *bhp* genes but was positive for the *aap*, *embp*, *atl*E, *aae*, *sdr*G/F, *clf*A, *eno* and *agr* genes. However, we were not able to type the *agr* locus.

## Discussion

We established a collaboration between animal and human health care professionals aimed at understanding if non-*aureus* staphylococci (NAS) responsible for human diseases share genetic similarities with those circulating in sheep, in consideration of the high number of dairy sheep farmed in the island and of the prominent role of these bacteria as mastitis agents.

A total of 195 NAS isolates, 125 from ovine mastitis and 70 from human clinical specimens, were analyzed for biofilm production and presence of autolysins, pyrogenic toxins, and MSCRAMM genes. We also typed *agr* alleles by PCR because the quorum sensing system regulates many virulence determinants involved in staphylococcal infections, including autolysins, adhesins, and toxins [19]. In sheep, the primary NAS detected were *S. epidermidis* followed by *S. chromogenes* and *S. haemolyticus*. At the same time, in humans we found primarily *S. haemolyticus* and *S. epidermidis*, followed by *S. lugdunensis*, *S. hominis*, *S. capitis*, *S. warneri*, *S. xylosus*, *S. pasteuri,* and *S. saprophyticus* subsp. *bovis*. *S. haemolyticus* has been associated with septicemia in neonates and skin infections; *S. epidermidis* is the main pathogen isolated in catheter-associated bloodstream infections (BSI); *S. lugdunensis* can cause acute endocarditis; *S. hominis* and *S. capitis* may induce BSI in neonates; *S. warneri* is associated with device-related bone and joint infections, while *S. pasteuri*, *S. xylosus* and *S. saprophyticus* subsp. *bovis* are not associated with a particular clinical infection, and their appearance as nosocomial pathogens could be related to previous contact with animals, mainly pig, cattle, sheep, and goats [26]; *S. epidermidis* represents the most frequently isolated species from ovine mastitis and human clinical specimens [1, 26, 27].

Overall, 65 NAS were able to form biofilm in vitro; however, the percentage of biofilm producers in sheep isolates was slightly lower than in human isolates. Moreover, we found a correlation between biofilm production and *ica* operon presence only in 5 *S. epidermidis* isolates, 4 human and 1 ovine. Some authors proposed to use this correlation as a pathogenesis marker to distinguish invasive from commensal isolates [28, 29]. However, we and others [11, 30, 31] demonstrated that PCR positivity for *ica*A/*ica*D genes can also be found in non-biofilm producers. Since the correlation between biofilm production and positivity for *ica*, *bhp*, *aap,* and *embp* genes is not clearly defined, we suggest considering all isolates that possess such genes as potentially invasive. In this work, only one *S. epidermidis* with these characteristics was isolated from ovine mastitis while the other 4 derived from catheters and blood. Noteworthy, a high positivity for the genes encoding the bifunctional adhesins/autolysins AtlE and Aae was found in both animal and human *S. epidermidis* isolates [3, 5]. In addition to bacteriolytic activity, AtlE and Aae act as adhesins by binding noncovalently to vitronectin and by causing the release of extracellular DNA (eDNA), a critical adherence/aggregation factor in biofilm formation [32]. The presence, of *atl*E and *aae* in *S. epidermidis* was accompanied by a high prevalence of *embp*, *sdr*G, *sdr*F, *eno* and *epb*S, all genes that mediate adherence to substrates containing fibronectin, fibrinogen, collagen, laminin and elastin, respectively [4, 33–35]. The ability of *S. epidermidis* to bind these substrates might represent a relevant mechanism by which it can adhere to and colonize different host sites. The *eno* gene was the only gene found in all NAS analyzed, except for *S. simulans*. In human NAS, the prevalence is 100%. Therefore, the ability of NAS to bind laminin, a major component of basal membrane of the vasculature, might play a possible role in to tissue invasion and blood dissemination.

The *agr* locus is a regulatory system that responds to host and environmental stimuli and controls the production of many virulence factors [24]. In *S. epidermidis*, three distinct *agr* groups have been recognized [25]. Li et al. [36] have linked the genetic polymorphism of the *agr* locus to pathogenicity; group-1_se_ was associated with pathogenicity, while healthy people mainly carried group-2se. In our human *S. epidermidis* isolates, *agr*-1_se_ was predominant (*n* = 17), followed by *agr*-2_se_ (*n* = 9). It is interesting to notice that almost all isolates possessing *ica* genes belonged to *agr*-1_se_. This may suggest a correlation of these virulence genes with a specific *agr* locus. However, other 8 *ica*A^−^/D^−^ isolates were present in the group-1_se_. The feature shared by all 17 isolates belonging to this group was the PCR positivity for the *atl*E, *aae*, *sdr*G, *eno* and *epb*S genes. On the other hand, among the 9 isolates grouped in the *agr*-2_se,_ 1 (from blood) was *ica*A^+^/D^+^, while the remaining ones were *ica*-negative. The common denominator of these 9 isolates was the PCR positivity for *aap*, *aae*, *sdr*G, *embp, eno,* and *epb*S. These findings suggest that the relationship between *agr* groups and *S. epidermidis* pathogenicity will require further investigation. As observed in our previous study [30], *agr*-3_se_ (*n* = 33) was predominant among ovine *S. epidermidis* isolates followed by *agr*-1_se_ (*n* = 21). These results may suggest a possible transmission of *S. epidermidis* isolates from the milkers to the ewes.

Unlike *S. chromogenes*, *S. haemolyticus, S. warneri and S. muscae* from ovine mastitis and *S. pasteuri* from human specimens, the other NAS were classified as WBP by the microplate adhesion technique but did not harbor *ica*A/D. According to Fredheim et al. [37], *S. haemolyticus* mainly produces a PIA-independent biofilm. However, we detected only the *aap* (2/17) and *embp* (1/17) genes in the present study by PCR. Only 4 human *S. haemolyticus* isolates possessed the *aap* gene coding a protein that mediates biofilm formation in strains lacking the *ica* genes [16]. Our data suggest that *ica* and *bhp* genes do not contribute significantly to *S. haemolyticus* biofilms’ protein components.

In *S. aureus* and in many other bacteria, toxins are critical contributors to aggressive virulence, even though *S. epidermidis* is not generally accepted as an enterotoxin producer [38, 39]. Based on our findings, the primary enterotoxin genes (*sea*, *seb*, *sec*, *sed* and *see*) and the *tsst*-1 gene were absent in all ovine and human NAS analyzed. On the contrary, Pedroso et al. [16] and Da Cunha et al. [40] detected high percentages of *sea* and *sec* genes in coagulase-negative staphylococci from hospitals of Brazil; also, Giormezis et al. [39] found a higher number of isolates positive for *tsst* among NAS from hospitals in Greece.

## Conclusion

In conclusion, we detected intercellular adhesion genes (*ica*AB) and other genes related to biofilm formation only in *S. epidermidis*, although we found *ica*A in ovine *S. caprae* and *S. saprophyticus*, and in human *S. capitis* and *S. xylosus*. The remaining isolates carried few virulence determinants. The ability to form biofilm observed in NAS isolates, especially *S. epidermidis*, might constitute a significant virulence factor facilitating colonization, infection, diffusion, and resistance.

## Methods

### Isolate collection

*Ovine isolates*: A total of 125 NAS were isolated from sheep milk samples that routinely arrive at the Istituto Zooprofilattico Sperimentale della Sardegna. Milk samples, collected from farms with mastitis problems in different provinces of Sardinia (Italy), were analyzed over 9 months (April-December 2017) (Table 1). The geographic distribution of these isolates is reported in Supplementary Fig. S1. Isolates were identified as: *S. epidermidis* (*n* = 57), *S. chromogenes* (*n* = 29), *S. haemolyticus* (*n* = 17), *S. simulans* (*n* = 8), *S. caprae* (*n* = 6), *S. warneri* (*n* = 5), *S. saprophyticus* (*n* = 1), *S. intermedius* (*n* = 1), and *S. muscae* (*n* = 1), by means of PCR-RFLP [27].

*Human isolates*: During the same period, 70 NAS isolates were collected from different clinical specimens at the microbiology laboratories of three major Sardinia hospitals. Isolates were anonymized without patient identifiers. Isolates and their origin are summarized in Table 1; 90% of the human NAS were recovered from hospitalized patients in intensive care unit, hematology, and orthopedics. The 70 isolates were identified by PCR-RFLP as *S. haemolyticus* (*n* = 28), *S. epidermidis* (*n* = 26), *S. lugdunensis* (*n* = 4), *S. hominis* (*n* = 4), *S. capitis* (*n* = 3), *S. warneri* (*n* = 2), *S. xylosus* (*n* = 1), *S. pasteuri* (*n* = 1), and *S. saprophyticus subsp. bovis* (*n* = 1) [27].

Statements of owner consent or patient consent were not required in this case since personal or sensitive data never accompanied samples. All Isolates were anonymized regarding the originating animal, flock, or patient, and were processed for phenotypic and molecular analyses without any original information linked to them.

### Phenotyping evaluation of biofilm production by the microtiter plate assay (MPA)

All 195 isolates were tested using the MPA technique, described by Vasileiou et al. [8] with some modifications. Briefly, a colony of each isolate was inoculated into a tube containing 1 mL Tryptone Soy Broth (TSB, Oxoid, Basingstoke, UK) for 16 h at 37 °C. Overnight culture was diluted 1:40 with TSB containing 0.25% glucose, and 200 μL per well were seeded in a sterile 96-well flat-bottomed microplate (Thermo Fisher, Rodano, IT) at 37 °C for 24 h. After three washes in PBS pH 7.4, the microplate was dried at 45 °C for 20 min, and wells were then stained with 1% crystal violet for 15 min at room temperature. After three washes with distilled water and subsequent drying at 45 °C for 20 min, 200 μL of 33% acetic acid were added to each well. Biofilm growth was measured at 630 nm in a microplate spectrophotometer (Multiskan GO, Thermo Fisher). Uninoculated wells containing TBS with glucose served as blanks. In each microplate, *S. epidermidis* ATCC 35984 and *S. epidermidis* ATCC 12228 were included as the positive and negative controls, respectively. Each isolate and both controls were tested in triplicate, and the assay was repeated two times at different dates. Isolates were classified into three categories based upon the median OD of isolates and positive and negative controls: biofilm-producing (OD isolate ≥ OD of the positive control), weak biofilm-producing (OD negative control < OD isolate < OD positive control) and non biofilm-producing (OD isolate ≤ negative control).

### Detection of biofilm, autolysins, MSCRAMMs and pyrogenic toxins genes

Genomic DNA was extracted from all 195 NAS isolates and Reference Strains (RS) according to Onni et al. [41]. Single-tube PCRs were performed for detecting genes related to biofilm production (*ica*A/D, *bhp*, *aap*, *embp*) [30, 31, 42, 43], autolysins (*atl*E and *aae*) [44, 45], MSCRAMMs (encoding clumping factor-*clf*A/B, fibronectin-binding protein-*fnb*A/B, encoding bone sialoprotein-binding protein-*bbp*, collagen-binding protein-*cna*, fibrinogen-binding protein-*fib*, laminin-binding protein-*eno*, elastin-binding protein-*ebp*S, and serine-aspartate repeat protein-*sdr*F/G) [19, 30, 46–49] and pyrogenic toxins (sea, seb, sec, sed, see and tsst-1) [50–52]. Primer sets are reported in Supplementary Table S1. PCR tests were carried out in a GeneAmp9700 DNA thermal cycler (Applied Biosystems, now Thermo Fisher Scientific, Waltham, MA, USA). The following RS were used as positive controls: *S. epidermidis* ATCC 35984 (*ica*A/D, *bhp*, *aap*, *embp, atl*E, *aae*, *sdr*F, *sdr*G), *S. aureus* ATCC 25923 (*clf*A/B, *bbp, epbs*), *S. aureus* ATCC 33591 (*fnb*A/B, *cna*, *fib, eno*), *S. aureus* ATCC 13565 (*sea*), *S. aureus* ATCC 14458 (*seb*), *S. aureus* ATCC19095 (*sec*), *S. aureus* ATCC 23235 (*sed*), *S. aureus* ATCC 27664 (*see*) and *S. aureus* ATCC 33586 (*tsst-1*).

### Typing of agr alleles

A 200 bp conserved region of the *agr* operon was amplified as described previously [19]. For isolate typing, we targeted *agr-1*_*se*_ to *agr-3*_*se*_ sequences [53]. As controls, we used *S. epidermidis* ATCC 35984 (*agr-1*_*se*_)*, S. epidermidis* isolate 1037 (*agr-2s*_*e*_) and *S. epidermidis* isolate 43,027 (*agr-3*_*se*_). Supplementary Table S1 reports primer sets and related references.

## Supplementary Information

**Additional file 1: Supplementary Table S1**. Oligonucleotide primers used for the detection of genes related to biofilm production (*ica*A/D*, bhp, aap* and *embp*), autolysins (*atl*E*, aae*), MSCRAMMs (*sdr*G, *sdr*F, *clf*A, *clf*B, *fnb*A, *fnb*B, *bbp*, *cna*, *fib, eno* and *epb*S), pyrogenic toxins (*sea*, *seb*, *sec*, *sed*, *see* and *tsst-1*) and for *agr* typing.

**Additional file 2: Supplementary Figure S1**. Map of Sardinia showing the location of all 125 non-*aureus* staphylococci isolates in every municipality. No copyright permission was required.

## Data Availability

All data supporting these research findings are included within the manuscript. The databases are available from the corresponding author upon request.

## Abbreviations

CRA: Congo Red Agar
MSCRAMMs: Microbial surface components recognizing adhesive matrix molecules
NAS: Non-aureus staphylococci
QS: Quorum-sensing system

## References

[1] Marogna G, Rolesu S, Lollai S, Tola S, Leori G (2010). Clinical findings in sheep farms affected by recurrent bacterial mastitis. Small Rumin Res.

[2] Onni T, Vidili A, Bandino E, Marogna G, Schianchi S, Tola S (2012). Identification of coagulase-negative staphylococci isolated from caprine milk samples by PCR-RFLP of groEL gene. Small Rumin Res.

[3] Martins KB, Faccioli PY, Bonesso MF, Fernandes S, Oliveira AA, Dantas A (2017). Characteristics of resistance and virulence factors in different species of coagulase-negative staphylococci isolated from milk of healthy sheep and animals with subclinical mastitis. J Dairy Sci.

[4] Vanderhaeghen W, Piepers S, Leroy F, Van Coillie E, Haesebrouck F, De Vliegher S (2015). Identification, typing, ecology and epidemiology of coagulase negative staphylococci associated with ruminants. Vet J.

[5] Von Eiff C, Peters G, Heilmann C (2002). Pathogenesis of infections due to coagulase-negative staphylococci. Lancet Infect Dis.

[6] Mack D, Davies AP, Harris LG, Rohde H, Horstkotte MA, Knobloch JK-M (2007). Microbial interactions in *Staphylococcus epidermidis* biofilms. Anal Bioanal Chem.

[7] Stepanovic S, Vukovic D, Dakic I, Savic B, Svabic-Vlahovic M (2000). A modified microtiter-plate test for quantification of staphylococcal biofilm formation. J Microbiol Methods.

[8] Vasileiou NGC, Chatzopoulos DC, Gougoulis DA, Sarrou S, Katsafadou AI, Spyrou V, Mavrogianni VS, Petinaki E, Fthenakis GC (2018). Slime-producing staphylococci as causal agents of subclinical mastitis in sheep. Vet Microbiol.

[9] Arciola CR, Campoccia D, Gamberini S, Cervellati M, Donati E, Montanaro L (2002). Detection of slime production by means of an optimised Congo red agar plate test based on a colourimetric scale in *Staphylococcus epidermidis* clinical isolates genotyped for *Ica* locus. Biomaterials..

[10] Kogan G, Sadovskaya I, Chaignon P, Chokr A, Jabbouri S (2006). Biofilms of clinical strains of *Staphylococcus* that do not contain polysaccharide intercellular adhesin. FEMS Microbiol Lett.

[11] Rohde H, Burandt EC, Siemssen N, Frommelt L, Burdelski C, Wurster S (2007). Polysaccharide intercellular adhesin or protein factors in biofilm accumulation of *Staphylococcus epidermidis* and *Staphylococcus aureus* isolated from prosthetic hip and knee joint infections. Biomaterials..

[12] Fey PD, Olson ME (2010). Current concepts in biofilm formation of *Staphylococcus epidermidis*. Future Microbiol.

[13] Heilmann C, Hussain M, Peters G, Götz F (1997). Evidence for autolysin-mediated primary attachment of *Staphylococcus epidermidis* to a polystyrene surface. Mol Microbiol.

[14] Heilmann C, Thumm G, Chhatwal GS, Hartleib J, Uekötter A, Peters G (2003). Identification and characterization of a novel autolysin (Aae) with adhesive properties from *Staphylococcus epidermidis*. Microbiology..

[15] Otto M (2012). Molecular basis of *Staphylococcus epidermidis* infections. Semin Immunopathol.

[16] Pei L, Flock JI (2001). Lack of *fbe*, the gene for a fibrinogen-binding protein from *Staphylococcus epidermidis*, reduces its adherence to fibrinogen coated surfaces. Microb Pathog.

[17] Arrecubieta C, Toba FA, Von Bayern M, Akashi H, Deng MC, Naka Y (2009). SdrF, a *Staphylococcus epidermidis* surface protein, contributes to the initiation of ventricular assist device driveline-related infections. PLoS Pathog.

[18] Trivedi S, Uhlemann AC, Herman-Bausier P, Sullivan SB, Sowash MG, Flores EY (2017). The surface protein SdrF mediates *Staphylococcus epidermidis* adherence to keratin. J Infect Dis.

[19] Pedroso SHSP, Sandes SHC, Luiz KCM, Dias RS, Filho RAT, Serufo JC (2016). Biofilm and toxin profile: a phenotypic and genotypic characterization of coagulase-negative staphylococci isolated from human bloodstream infections. Microb Pathog.

[20] Balaban N, Rasooly A (2000). Staphylococcal enterotoxins. Int J Food Microbiol.

[21] Boerema JA, Clemens R, Brightwell G (2006). Evaluation of molecular methods to determine enterotoxigenic status and molecular genotype of bovine, ovine, human and food isolates of *Staphylococcus aureus*. Int J Food Microbiol.

[22] Thomas DY, Jarraud S, Lemercier B, Cozon G, Echasserieau K, Etienne J (2006). Staphylococcal enterotoxin-like toxins U2 and V, two new staphylococcal superantigens arising from recombination within the enterotoxin gene cluster. Infect Immun.

[23] Chan WC, Coyle BJ, Williams P (2004). Virulence regulation and *quorum sensing* in staphylococcal infections: competitive AgrC antagonists as quorum sensing inhibitors. J Med Chem.

[24] Kong KF, Vuong C, Otto M (2006). Staphylococcus quorum sensing in biofilm formation and infection. Int J Med Microbiol.

[25] Dufour P, Jarraud S, Vandenesch F, Greenland T, Novick RP, Bes M (2002). High genetic variability of the *agr* locus in *Staphylococcus* species. J Bacteriol.

[26] Becker K, Heilmann C, Peters G (2014). Coagulase-negative staphylococci. Clin Microbiol Rev.

[27] Turchi B, Bertelloni F, Marzoli F, Cerri D, Tola S, Azara E (2020). Coagulase negative staphylococci from ovine milk: genotypic and phenotypic characterization of susceptibility to antibiotics, disinfectants and biofilm production. Small Rumin Res.

[28] Frebourg NB, Lefebvre S, Baert S, Lemeland JF (2000). PCR-based assay for discrimination between invasive and contaminating *Staphylococcus epidermidis* strains. J Clin Microbiol.

[29] Mekni MA, Bouchami O, Achour W, Ben HA (2012). Strong biofilm production but not adhesion virulence factors can discriminate between invasive and commensal *Staphylococcus epidermidis* strains. APMIS..

[30] Abbondio M, Fois I, Longheu C, Azara E, Tola S (2019). Biofilm production, quorum sensing system and analysis of virulence factors of *Staphylococcus epidermidis* collected from sheep milk samples. Small Rumin Res.

[31] Salgueiro VC, Iorio NLP, Ferreira MC, Chamon RC, Dos Santos KRN (2017). Methicillin resistance and virulence genes in invasive and nasal *Staphylococcus epidermidis* isolates from neonates. BMC Microbiol.

[32] Qin Z, Ou Y, Yang L, Zhu Y, Tolker-Nielsen T, Molin S (2007). Role of autolysin-mediated DNA release in biofilm formation of *Staphylococcus epidermidis*. Microbiology..

[33] Christner M, Franke GC, Schommer NN, Wendt U, Wegert K, Pehle P (2010). The giant extracellular matrix-binding protein of *Staphylococcus epidermidis* mediates biofilm accumulation and attachment to fibronectin. Mol Microbiol.

[34] Carneiro CRW, Postol E, Nomizo R, Reis LFL, Brentani RR (2004). Identification of enolase as a laminin-binding protein on the surface of *Staphylococcus aureus*. Microb Infect.

[35] Downer R, Roche F, Park PW, Mecham RP, Foster TJ (2002). The elastin-binding protein of *Staphylococcus aureus* (EbpS) is expressed at the cell surface as an integral membrane protein and not as a cell wall-associated protein. J Biol Chem.

[36] Li M, Guan M, Jiang XF, Yuan FY, Xu M, Zhang WZ (2004). Genetic polymorphism of the accessory gene regulator (*agr*) locus in *Staphylococcus epidermidis* and its association with pathogenicity. J Med Microbiol.

[37] Fredheim EGA, Klingenberg C, Rohde H, Frankenberger S, Gaustad P, Flægstad T (2009). Biofilm formation by *Staphylococcus haemolyticus*. J Clin Microbiol.

[38] Otto M (2009). *Staphylococcus epidermidis* - the “accidental” pathogen. Nat Rev Microbiol.

[39] Giormezis N, Kolonitsiou F, Foka A, Drougka E, Liakopoulos A, Makri A (2014). Coagulase-negative staphylococcal bloodstream and prosthetic-device-associated infections: the role of biofilm formation and distribution of adhesin and toxin genes. J Med Microbiol.

[40] Da Cunha MDLRS, Calsolari RAO, Araújo JP (2007). Detection of enterotoxin and toxic shock syndrome toxin 1 genes in *Staphylococcus*, with emphasis on coagulase-negative staphylococci. Microbiol Immunol.

[41] Onni T, Sanna G, Larsen J, Tola S (2011). Antimicrobial susceptibilities and population structure of *Staphylococcus epidermidis* associated with ovine mastitis. Vet Microbiol.

[42] Vandecasteele SJ, Peetermans WE, Merckx RR, Rijnders BJA, Van Eldere J (2003). Reliability of the Ica, aap and atlE genes in the discrimination between invasive, colonizing and contaminant *Staphylococcus epidermidis* isolates in the diagnosis of catheter-related infections. Clin Microbiol Infect.

[43] Gu J, Li H, Li M, Vuong C, Otto M, Wen Y (2005). Bacterial insertion sequence IS256 as a potential molecular marker to discriminate invasive strains from commensal strains of *Staphylococcus epidermidis*. J Hosp Infect.

[44] Møretrø T, Hermansen L, Holck AL, Sidhu MS, Rudi K, Langsrud S (2003). Biofilm formation and the presence of the intercellular adhesion locus *Ica* among staphylococi from food and food processing environments. Appl Environ Microbiol.

[45] Lou Q, Zhu T, Hu J, Ben H, Yang J, Yu F (2011). Role of the SaeRS two-component regulatory system in *Staphylococcus epidermidis* autolysis and biofilm formation. BMC Microbiol.

[46] Stephan R, Annemüller C, Hassan AA, Lämmler C (2001). Characterization of enterotoxigenic *Staphylococcus aureus* strains isolated from bovine mastitis in north-East Switzerland. Vet Microbiol.

[47] Peacock SJ, Moore CE, Justice A, Kantzanou M, Story L, Mackie K (2002). Virulent combinations of adhesin and toxin genes in natural populations of *Staphylococcus aureus*. Infect Immun.

[48] Vancraeynest D, Hermans K, Haesebrouck F (2004). Genotypic and phenotypic screening of high and low virulence *Staphylococcus aureus* isolates from rabbits for biofilm formation and MSCRAMMs. Vet Microbiol.

[49] Tristan A, Ying L, Bes M, Etienne J, Vandenesch F, Lina G (2003). Use of multiplex PCR to identify *Staphylococcus aureus* adhesins involving in human hematogenous infection. J Clin Microbiol.

[50] Monday SR, Bohach GA (1999). Use of multiplex PCR to detect classical and newly described pyrogenic toxin genes in staphylococcal isolates. J Clin Microbiol.

[51] Johnson WM, Tyler SD, Ewan EP, Ashton FE, Pollard DR, Rozee KR (1991). Detection of genes for enterotoxins, exfoliative toxins, and toxic shock syndrome toxin 1 in *Staphylococcus aureus* by the polymerase chain reaction. J Clin Microbiol.

[52] Løvseth A, Loncarevic S, Berdal KG (2004). Modified multiplex PCR method for detection of pyrogenic exotoxin genes in staphylococcal isolates. J Clin Microbiol.

[53] Lina G, Boutite F, Tristan A, Bes M, Etienne J, Vandenesch F (2003). Bacterial competition for human nasal cavity colonization: role of staphylococcal *agr* alleles. Appl Environ Microbiol.

