# Supplementary material for: Comparative profiling of agr locus, virulence, and biofilm-production genes of human and ovine non-aureus staphylococci
Source: BMC Vet Res. 2022 Jun 2;18:212. doi: 10.1186/s12917-022-03257-w (PMC9161600; doi:10.1186/s12917-022-03257-w)
Supplement: Supplementary file 1 — Additional file 1: Supplementary Table S1. Oligonucleotide primers used for the detection of genes related to biofilm production (icaA/D, bhp, aap and embp), autolysins (atlE, aae), MSCRAMMs (sdrG, sdrF, clfA, clfB, fnbA, fnbB, bbp, cna, fib, eno and epbS), pyrogenic toxins (sea, seb, sec, sed, see and tsst-1) and for agr typing. [file 12917_2022_3257_MOESM1_ESM.docx]

**Supplementary Table S1** Oligonucleotide primers used for the detection of genes related to biofilm production (*ica*A/D*, bhp, aap* and *embp*), autolysins (*atl*E*, aae*), MSCRAMMs (*sdr*G, *sdr*F, *clf*A, *clf*B, *fnb*A, *fnb*B, *bbp*, *cna*, *fib,* *eno* and *epb*S), pyrogenic toxins (*sea*, *seb*, *sec*, *sed*, *see* and *tsst-1*) and for *agr* typing

| **Gene** | **Nucleotide sequence (5'-3')** | **Amplicon size (bp)** |
| --- | --- | --- |
| **Biofilm production** |  |  |
| *icaA* | CACGTGCTCTATGCTGGATG  CCGTTGGATATTGCCTCTGT | 502 |
| *icaD* | AGAGGCAATATCCAACGGT  TCATATGTCACGACCTTTCT | 290 |
| *bhp* | TGGTATTAGGAAGCTCTCA  ATACCAGCGTGACGCAAATC | 935 |
| *aap* | ATACAACTGGTGCAGATGGTTG  GTAGCCGTCCAAGTTTTACCAG | 399 |
| *embp* | AGCGGTACAAATGTCAATATC  AGAAGTGCTCTAGCATCATCC | 455 |
| **Autolysins** |  |  |
| *atlE* | CAACTGCTCAACCGAGAACA  TTTGTAGATGTTGTGCCCCA | 682 |
| *aae* | AACAAATTGATAAAGCAACG  GTTGTCTTTCCTTTAGTGTC | 216 |
| **MSCRAMMS** |  |  |
| *sdrG/fbe* | CTACAAGTTCAGGTCAAGGACAAGG  GCGTCGGCGTATATCCTTCAG | 273 |
| *sdrF* | GCTGAAGACAATCAATTAG  TTGTCTCTAACTGCATTTG | 750 |
| *clfA* | GGCTTCAGTGCTTGTAGG  TTTTCAGGGTCAATATAAGC | 1042 |
| *clfB* | TGCAAGATCAAACTGTTCCT  TCGGTCTGTAAATAAAGGTA | 596 |
| *fnbA* | CACAACCAGCAAATATAG  CTGTGTGGTAATCAATGTC | 1362 |
| *fnbB* | GTAACAGCTAATGGTCGAATTGATACT  CAAGTTCGATAGGAGTACIATGTTC | 524 |
| *bbp* | CAGTAAATGTGTCAAAGA  TACACCCTGTTGAACTG | 1050 |
| *cna* | GTCAAGCAGTTATTAACACCAGAC  AATCAGTAATTGCATTTGTCCACTG | 423 |
| *eno* | ACGTGCAGCAIGCTGACT  CAACAGCATYCTTCAGTACCTTC | 302 |
| *fib* | CTACAACTACAATTGCCGTCAACAG  GCTCTTGTAAGACCATTTTCTTCAC | 404 |
| *epbS* | AGAATGCTTTTGCAATGGAT  AATATCGCTAATGCACCGAT | 652 |
| **Pyrogenic toxins** |  |  |
| *sea* | GCAGGGAACAGCTTTAGGC  GTTCTGTAGAAGTATGAAACACG | 520 |
| *seb* | ACATGTAATTTTGATATTCGCACTG  TGCAGGCATCATGTCATACCA | 667 |
| *sec* | CTTGTATGTATGGAGGAATAACAA  TGCAGGCATCATATCATACCA | 283 |
| *sed* | CTAGTTTGGTAATATCTCCT  TAATGCTATATCTTATAGGG | 317 |
| *see* | TACCAATTAACTTGTGGATAGAC  CTCTTTGCACCTTACCGC | 170 |
| *tsst-1* | GCTTGCGACAACTGCTACAG  TGGATCCGTCATTCATTGTTAT | 559 |
| ***agr* locus** |  |  |
| *agr* | CATAGCACTGAGTCCAAGGA  CAATCGGTGACTTAGTAAAATG | 200 |
| *agr-1se* | GGCATTAGTCGGATTAATTATTACG  TGTAGGCCTGCAAACGG | 438 |
| *agr-2se* | TTTACCATTTGCAGCTATACAAGTG  ATAACAATAATATAACCAAACTCAAAAGTACAG | 575 |
| *agr-3se* | GAAAGAGTGTATTCAATGGATGAGC  TAAATATTATGTATTATATCTTCAGTATATAAAGAGATGA | 338 |
