# Supplementary material for: Comparative profiling of agr locus, virulence, and biofilm-production genes of human and ovine non-aureus staphylococci
Source: BMC Vet Res. 2022 Jun 2;18:212. doi: 10.1186/s12917-022-03257-w (PMC9161600; doi:10.1186/s12917-022-03257-w)

**Supplementary Figure S1** Map of Sardinia showing the location of all 125 non-*aureus* staphylococci isolates in every municipality. No copyright permission was required.


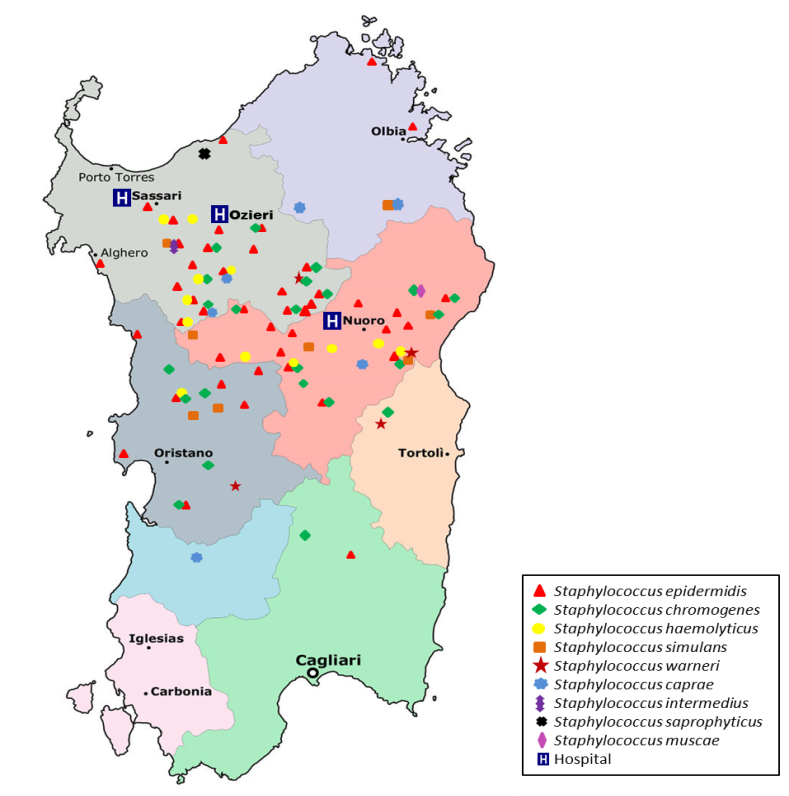

Supplement: Supplementary file 2 — Additional file 2: Supplementary Figure S1. Map of Sardinia showing the location of all 125 non-aureus staphylococci isolates in every municipality. No copyright permission was required. [file 12917_2022_3257_MOESM2_ESM.docx]
